# Younger, Lighter, Drier: The Impact of Age and Body Mass Index on Treatment Success in Pediatric Monosymptomatic Nocturnal Enuresis

**DOI:** 10.3390/children13020241

**Published:** 2026-02-09

**Authors:** Gokhan Demirtas, Gunay Ekberli, Suleyman Tagci, Huseyin Tugrul Tiryaki

**Affiliations:** 1 Department of Pediatric Urology, Sincan Training and Research Hospital, Ankara 06930, Turkey; drgokhandemirtas@gmail.com; 2 Department of Pediatric Urology, Ankara Bilkent City Hospital, Ankara 06800, Turkey; suleyman_tagci@hotmail.com (S.T.); httiryaki@hotmail.com (H.T.T.)

**Keywords:** primary monosymptomatic nocturnal enuresis, age, body mass index, desmopressin therapy, treatment success

## Abstract

**Highlights:**

**What are the main findings?**

- Younger age and lower body mass index are independently associated with higher treatment success in pediatric monosymptomatic nocturnal enuresis.
- Overweight and older children show significantly reduced response rates to standard first-line therapies.

**What are the implications of the main findings?**

- Age and body mass index should be considered when counseling families and setting realistic expectations for treatment outcomes.
- Early intervention and weight-aware management strategies may improve therapeutic success in pediatric nocturnal enuresis.

**Abstract:**

Objectives: To evaluate the effects of age, body mass index, and treatment duration on treatment response in children with primary monosymptomatic nocturnal enuresis and to determine the contribution of these variables to clinical outcomes. Methods: Data from 560 pediatric patients treated with desmopressin due to primary monosymptomatic nocturnal enuresis were retrospectively analyzed. Patient demographics, body mass index classifications, early treatment response, and dryness rates at 3 and 6 months were evaluated. Categorical variables were analyzed using the chi-square or Fisher’s exact test, and continuous variables were evaluated using Spearman correlation analysis. Significant factors predicting treatment success were investigated using multivariable analyses. Results: Treatment success was significantly higher in younger age groups, with increased early response rates and shorter treatment duration (*p* < 0.001). Higher body mass index was associated with a delay in early treatment response and the need for longer treatment (*p* < 0.0001). While the likelihood of treatment success decreased with increasing age, higher body mass index demonstrated a modest positive association with treatment response after multivariable adjustment. Conclusions: The findings indicate that age and body mass index are important determinants of treatment response in pediatric monosymptomatic nocturnal enuresis. These results suggest that treatment strategies should be individualized by considering both age and body mass index.

## 1. Introduction

Nocturnal enuresis is one of the most prevalent lower urinary tract disorders in childhood and carries a notable psychosocial burden affecting both the child and the family [1]. According to the International Children’s Continence Society (ICCS), primary monosymptomatic nocturnal enuresis (PMNE) is defined as involuntary urine loss during sleep occurring at least once per week for a minimum of three consecutive months in children aged five years or older, in the absence of daytime lower urinary tract symptoms [1]. Although generally considered a benign condition with a tendency toward spontaneous remission, its underlying pathophysiology is multifactorial and remains incompletely elucidated. Current evidence highlights mechanisms such as nocturnal polyuria, reduced functional bladder capacity, elevated arousal threshold, and, in some patients, detrusor overactivity [1,2]. Age has consistently been identified as an important determinant of therapeutic response. Younger children typically demonstrate faster and more favorable treatment outcomes, supporting the rationale for age-adapted management strategies [1]. Although sex-related differences have been reported—often characterized by delayed attainment of dryness in boys—the literature remains inconsistent, and their independent clinical relevance is uncertain [3,4].

Parallel to the global rise in pediatric obesity, increasing attention has been directed toward body mass index (BMI) as a potential modifier of both the prevalence and clinical course of MNE. Several studies have documented higher rates of enuresis among overweight and obese children and suggested an association between elevated BMI and reduced treatment responsiveness; however, the extent to which BMI influences overall treatment success remains incompletely defined [5,6,7,8]. Proposed mechanisms include alterations in nocturnal urine production, impaired arousal pathways, sleep-disordered breathing, and potential reductions in functional bladder capacity [9].

The presented study aims to evaluate the impact of age, sex, and BMI on treatment success in children with monosymptomatic nocturnal enuresis and to identify clinically meaningful determinants that may inform individualized therapeutic approaches.

## 2. Materials and Methods

### 2.1. Study Population

A total of 560 children treated with desmopressin due to PMNE and followed up in our clinic between January 2024 and June 2025 were included in the study. Patients were classified according to sex (female, male), age groups (6–8, 8–10, 10–12, >12 years), and BMI values. Body mass index values were categorized as “normal weight,” “overweight,” and “obese” according to the percentile curves defined by the World Health Organization (WHO) for children.

Due to the retrospective nature of the study, objective measurements for nocturnal polyuria (such as nocturnal urine volume or osmolality) were not systematically available; therefore, patients were not subclassified according to underlying pathophysiological mechanisms.

### 2.2. Treatment Protocol

The initial desmopressin dose was 120 µg in children with normal BMI, whereas a starting dose of 240 µg was selected for overweight and obese children based on clinical considerations, including potentially higher nocturnal diuresis and reduced arousal. When necessary, titration to 240 µg was permitted after two weeks. All patients and families received standard supportive advice, including lifestyle counseling and fluid intake regulation in the hours before sleep, simultaneously with medical treatment, in accordance with routine clinical practice.

The treatment period was standardized as 3 months, 6 months, or longer than 6 months. The presence of an initial treatment response was defined as achieving nighttime dryness within the first two months after initiation of therapy. If no dryness occurred, or if dryness was observed only rarely, the initial response was classified as “absent.”

Treatment success was defined as having at most one wet night per week or being completely dry by the end of treatment. Patients with more than one wet night per week after treatment were classified as “unsuccessful.”

### 2.3. Follow-Up

At the 3rd and 6th month follow-ups, enuresis status was assessed. Patients who had one or more wet nights per week were classified as “wetting present,” and those with fewer than one wet night per week were classified as “no wetting.”

### 2.4. Statistical Analyses

Statistical analyses were performed using IBM SPSS Statistics for Windows, Version 26.0 (IBM Corp., Armonk, NY, USA). Categorical variables were evaluated using the Chi-square test or Fisher’s exact test when expected cell counts were low. Correlations between continuous variables and treatment outcomes were assessed with Spearman’s rank correlation analysis. Independent predictors of treatment success were examined using a binary logistic regression model. A *p*-value < 0.05 was considered statistically significant.

## 3. Results

A total of 560 children treated with desmopressin due to PMNE were included in the study. Of these, 196 (35%) were female and 364 (65%) were male. The majority of patients were aged 6–8 years, followed by the 8–10, 10–12, and >12-year age groups. According to BMI classification, 307 children were of normal weight, 179 were overweight, and 74 were obese.

Treatment success differed significantly according to sex (*p* = 0.0021), with higher success rates observed in males than in females. Treatment success also varied significantly across age groups (*p* < 0.0001), with the highest success rates observed in children aged 6–8 years and a progressive decline with increasing age (Table 1).

Early treatment response differed significantly across age groups (*p* < 0.0001). Early response within the first two months was most frequently observed in children aged 6–8 years and decreased markedly with increasing age (Table 2).

Dryness rates at the 3- and 6-month follow-ups differed significantly across age groups (*p* < 0.0001). At both time points, younger children demonstrated higher dryness rates compared with older age groups, with the highest rates observed among children aged 6–8 years (Table 3).

Treatment duration demonstrated a clear age-dependent pattern, with progressively longer treatment courses observed in older age groups (*p* < 0.0001). Notably, none of the children older than 12 years received short-term treatment, whereas extended treatment durations were more frequently required among older patients (Table 4).

When treatment response patterns were evaluated according to desmopressin dose, early treatment response was more frequently observed in patients receiving the lower dose. However, dryness rates at six months were comparable between the dose groups (Table 5). Notably, the vast majority of patients in the high-dose group were overweight or obese.

Treatment success differed significantly across BMI categories (*p* = 0.038), with higher success rates observed in overweight children compared with those of normal weight (Table 6). No significant differences were observed between normal-weight and obese children or between overweight and obese children. Early treatment response within the first two months decreased progressively with increasing BMI (*p* < 0.0001) (Table 6).

At the 3-month follow-up, nighttime dryness rates did not differ among BMI categories. In contrast, at the 6-month follow-up, higher dryness rates were observed in overweight and obese children compared with those in the normal-weight group (Table 7).

Treatment duration differed significantly across BMI categories (*p* = 0.006). Children with normal BMI were more frequently treated for shorter durations, whereas overweight and obese children more commonly required longer treatment courses (Table 8).

### Logistic Regression Analysis

A multivariable binary logistic regression analysis was performed to identify independent predictors of treatment success. Age, BMI category, and treatment duration were identified as independent predictors of treatment outcome, whereas sex was not a significant predictor (Table 9).

Model characteristics: number of observations = 446; Pseudo R^2^ = 0.53; overall model *p*-value < 0.001. Abbreviations: BMI, body mass index; OR, odds ratio; CI, confidence interval.

## 4. Discussion

In this study, we examined the influence of age, sex, and BMI on treatment outcomes in 560 children with PMNE. Our findings indicate that younger age was associated with higher treatment success, whereas increasing BMI correlated with prolonged treatment duration and delayed initial response. Male patients showed a higher crude success rate in univariate analyses; however, this association disappeared after adjustment for age, BMI, and treatment duration in the multivariable logistic regression model, indicating that sex was not an independent predictor of treatment outcome.

The impact of age on therapeutic success in monosymptomatic nocturnal enuresis remains a subject of debate in the literature. The pathophysiological framework proposed by Nørgaard and colleagues—comprising increased nocturnal urine production, reduced functional bladder capacity, and an elevated arousal threshold—continues to represent the most widely accepted model explaining nocturnal enuresis [10]. Although neuropsychological maturation generally progresses with age, some children may retain a high arousal threshold and insufficient functional bladder capacity adaptation, which may limit treatment responsiveness. This conceptual framework provides a mechanistic basis for the observation that younger children often respond more favorably to therapy.

Existing literature presents inconsistent findings regarding the role of age. Van Herzeele et al. reported an age-related increase in response to desmopressin treatment [11], whereas Topalovic et al. identified younger age (<7 years) as a predictor of relapse rather than initial treatment failure [12]. In contrast, studies reporting that age does not independently predict treatment success also exist [13]. These discrepancies may reflect variations in age distribution, baseline wet-night frequency, treatment protocols, and behavioral adherence across study populations. Furthermore, considering the natural history of nocturnal enuresis—where approximately 15% of affected children achieve spontaneous remission each year [14,15], older children with persistent symptoms may represent a distinct and more treatment-resistant clinical phenotype rather than a simple delay in maturation.

In our cohort, age emerged as a significant and independent determinant of treatment outcome. Children aged 6–8 years demonstrated markedly higher success rates, faster initial response, shorter overall treatment duration, and higher dryness rates at the third month. These findings support the concept that enhanced neurodevelopmental plasticity and behavioral adaptability in early childhood may facilitate faster and more durable therapeutic responses, underscoring the clinical value of age-tailored treatment strategies.

Liu et al. demonstrated that age, sex, body weight, bladder capacity, nocturnal polyuria, and baseline wet-night frequency did not predict desmopressin dose requirements and concluded that optimal dosing should be individualized according to clinical response rather than demographic or physiological parameters [16].

In the present study, six-month success rates were comparable between patients initially treated with low-dose and high-dose desmopressin. Although low-dose therapy yielded higher dryness rates at three months, this difference had disappeared by month six. These findings indicate that early differences in response between dose groups may diminish over time when treatment is appropriately adjusted and maintained.

Obesity has increasingly been recognized as a clinically meaningful risk factor for nocturnal enuresis, largely due to its rising prevalence in childhood and its multifactorial metabolic and urological consequences [17,18,19]. Dysregulation of antidiuretic hormone secretion, disturbances in fluid–electrolyte balance, and alterations in sleep architecture may collectively contribute to increased nocturnal urine output and elevated arousal thresholds. Reports of reduced functional bladder capacity, irregular voiding patterns, and poorer adherence to behavioral interventions among obese children further support a pathophysiological predisposition to more persistent enuretic symptoms [19]. Wang et al. demonstrated a significant association between obesity and urinary urgency—though not nocturnal enuresis—highlighting the heterogeneous nature of BMI-related lower urinary tract effects [20]. In our cohort, overweight and obese children comprised nearly 45% of all cases, consistent with previous studies identifying elevated BMI as a relevant risk factor for enuresis.

Evidence also suggests that obesity may influence treatment outcomes. Ma et al. reported reduced therapeutic response among obese children [19], whereas Güven et al. found significantly higher success rates in children below the 85th BMI percentile [21]. These findings underscore the need to consider BMI as not only a potential etiological factor but also a determinant of therapeutic stratification.

In our study, the predominance of overweight and obese children in the high-dose desmopressin group was associated with slower early treatment response and longer treatment durations, reflecting a more resistant initial enuretic profile. This delayed early responsiveness aligns with prior reports indicating that elevated BMI may negatively affect initial treatment outcomes. Consistent with this pattern, early response was highest among normal-BMI children (22.5%) and declined stepwise in overweight (15.6%) and obese (10.8%) patients, supporting the notion that increased BMI adversely influences the early phase of therapy. However, despite their poorer early response, overweight and obese children demonstrated the highest six-month success rates (96.6% and 97.3%, respectively), surpassing normal-weight peers (90.9%). This paradox is best explained by treatment dynamics rather than intrinsic physiological advantage: children with elevated BMI were significantly more likely to receive higher desmopressin doses and prolonged therapy, allowing sufficient cumulative exposure for delayed responders to achieve dryness. Thus, elevated BMI appears to influence the timing of treatment response rather than the overall likelihood of treatment success, emphasizing the importance of adequate dosing, adherence, and extended treatment duration in managing enuresis among children with higher BMI.

Multivariable logistic regression analysis identified age as an independent negative predictor of treatment success, whereas BMI demonstrated an unexpected independent positive association. The age-related decline in treatment success aligns with previous evidence indicating greater therapeutic resistance and reduced adherence among older enuretic children. In contrast, the positive association between BMI and treatment outcome should be interpreted cautiously. This finding is likely influenced by sample characteristics, interactions between age and BMI, and differential treatment patterns—particularly the tendency for overweight and obese children to receive higher desmopressin doses and prolonged therapy. The inverse association observed between treatment duration and success most likely reflects clinical practice patterns, wherein children with slower or insufficient early response remain in therapy for extended periods until improvement is achieved. Collectively, these results highlight the complex interplay between age, BMI, and treatment duration, emphasizing the need for individualized therapeutic planning.

## 5. Conclusions

This study demonstrates that age, BMI status, and treatment duration are the principal determinants of therapeutic success in monosymptomatic nocturnal enuresis. Younger children exhibited the most favorable clinical profile, characterized by higher success rates and more rapid treatment response, whereas elevated BMI was associated with delayed early improvement and the need for longer treatment courses. Despite this delay, children with higher BMI ultimately achieved excellent long-term outcomes when therapy was adequately dosed and sustained. Increasing age consistently reduced the likelihood of treatment success, whereas BMI functioned as a clinical modifier that influenced the timing—rather than the overall probability—of therapeutic response. From a clinical perspective, these findings support an individualized approach to desmopressin therapy in children with monosymptomatic nocturnal enuresis. Younger patients may be expected to achieve treatment success more rapidly, while children with higher BMI may require prolonged treatment despite a slower initial response. Awareness of this pattern may help clinicians tailor follow-up strategies, improve adherence, and provide more accurate counseling to families, thereby avoiding premature discontinuation of therapy and minimizing overall treatment outcomes.

### Strengths and Limitations

The primary strengths of this study include its relatively large sample size and the incorporation of BMI as a key clinical variable, allowing a nuanced evaluation of its role in treatment response. However, the retrospective design introduces inherent limitations, including potential selection bias and incomplete control of confounding factors. In addition, the absence of objective measurements such as urine osmolality, nocturnal polyuria assessment, and formal sleep studies restricts the ability to fully elucidate the underlying mechanisms contributing to variations in treatment outcomes. These limitations should be considered when interpreting the findings.

## Figures and Tables

**Table 1:** Distribution of treatment success across age groups.

Age Group (Years)	Successful (n)	Unsuccessful (n)	Success Rate (%)
6–8 years	304	6	98.1
8–10 years	136	13	91.3
10–12 years	50	13	79.4
>12 years	22	16	57.9

**Table 2:** Early treatment response across age groups.

Age Group (Years)	Early Response/Total	Percentage (%)
6–8 years	72/310	23.2
8–10 years	30/149	20.1
10–12 years	2/63	3.2
>12 years	1/38	2.6

**Table 3:** Dryness rates at 3 and 6 months according to age groups.

Age Group (Years)	Dry at 3 Months n (%)	Dry at 6 Months n (%)
6–8 years	283/310 (91.3%)	298/310 (96.1%)
8–10 years	100/149 (67.1%)	136/149 (91.3%)
10–12 years	55/63 (87.3%)	61/63 (96.8%)
>12 years	26/38 (68.4%)	29/38 (76.3%)

**Table 4:** Treatment duration according to age groups.

Age Group (Years)	≤3 Months n (%)	>3 Months n (%)	>6 Months n (%)	Total (n)
6–8 years	120 (38.7%)	150 (48.4%)	40 (12.9%)	310
8–10 years	65 (43.6%)	70 (47.0%)	14 (9.4%)	149
10–12 years	12 (19.0%)	30 (47.6%)	21 (33.3%)	63
>12 years	0 (0%)	14 (36.8%)	24 (63.2%)	38

**Table 5:** Early treatment response and dryness rates at 3 and 6 months according to desmopressin dose.

Desmopressin Dose	n	Early Response (%)	Dry at 3 Months (%)	Dry at 6 Months (%)
120 µg	261	24.5	87	92
240 µg	299	13.7	79.3	95

**Table 6:** Treatment success and early response according to BMI categories.

BMI Category	n	Successful n (%)	Unsuccessful n (%)	Early Response n (%)
Normal	307	272 (88.6%)	35 (11.4%)	69 (22.5%)
Overweight	179	172 (96.1%)	7 (3.9%)	28 (15.6%)
Obese	74	68 (91.9%)	6 (8.1%)	8 (10.8%)

**Table 7:** Nighttime dryness rates at 3- and 6-month follow-up according to BMI categories.

BMI Category	Total (n)	Dry at 3 Months n (%)	Dry at 6 Months n (%)
Normal	307	259 (84.4%)	279 (90.9%)
Overweight	179	145 (81.0%)	173 (96.6%)
Obese	74	60 (81.1%)	72 (97.3%)

**Table 8:** Treatment duration according to BMI categories.

BMI Category	3 Months	6 Months	Longer	Total (n)
Normal	165	115	27	307
Overweight	80	74	25	179
Obese	21	40	13	74

**Table 9:** Multivariable binary logistic regression analysis of treatment success.

Variable	OR	95% CI	*p*-Value
Age (per year increase)	0.16	0.09–0.28	<0.001
BMI category (increase)	21.02	4.97–89.14	<0.001
Male (sex)	0.53	0.19–1.49	0.23
Treatment duration (per month)	5.87	2.98–11.58	<0.001

## Data Availability

The original contributions presented in this study are included in the article. Further inquiries can be directed to the corresponding author.

## Abbreviations

The following abbreviations are used in this manuscript:

PMNE	Primary monosymptomatic nocturnal enuresis
BMI	Body mass index
ICCS	International Children’s Continence Society
WHO	World Health Organization
OR	Odds ratio
CI	Confidence interval
µg	Microgram
SPSS	Statistical Package for the Social Sciences
